# Ixekizumab successfully treated severe pityriasis rubra pilaris after COVID‐19 vaccination

**DOI:** 10.1002/ski2.139

**Published:** 2022-06-16

**Authors:** Ya‐ting Liu, Shang‐shang Wang

**Affiliations:** ^1^ Department of Dermatology Huashan Hospital Fudan University Shanghai China

## Abstract

Pityriasis rubra pilaris is an inflammatory dermatologic disorder of unknown cause. We report a 67‐year‐old man with Pityriasis rubra pilaris might induced by COVID‐19 vaccination. The patient developed the lesions after the first dose of vaccine and significantly aggravated after the second dose. He had poor effect and liver function impairment developed after acitretin used, but achieved satisfactory efficacy after replacement to ixekizumab, an interleukin‐17A inhibitor.

## Graphical abstract

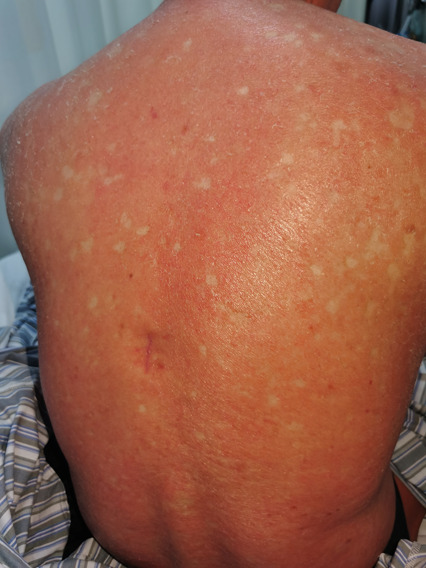

## 1 INTRODUCTION

Pityriasis rubra pilaris (PRP) is a rare papulo‐squamous dermatosis of uncertain aetiology. Pityriasis rubra pilaris has a variable clinical presentation, Type I (classical adult) presents with diffuse erythroderma with island of sparing and orange‐red palmo‐plantar keratoderma.^1^ During the COVID‐19 pandemic, SARS CoV‐2 vaccination has played an important role in management of the outbreak and a great number of people around the world have received various types of COVID‐19 vaccine. We contributed our experience with a new case of refractory PRP in adults after COVID‐19 vaccination, and were treated with ixekizumab, with a good response within 4 weeks of treatment.

## 2 CASE REPORT

A 67‐year‐old Chinese man presented with generalised erythematous plaques and scaly eruption 7 days after the second dose of an inactivated COVID‐19 vaccine (CoronaVac). He developed facial erythema with mild pruritus 12 days after he received the first dose of COVID‐19 vaccine and was administered cetirizine tablets together with topical calcineurin inhibitors but no significant improvement was observed. After the second dose of COVID‐19 vaccine, lesions progressed rapidly, covering almost 90% of his integument. He had a previous history of type 2 diabetes and fatty liver, and was treated with subcutaneous insulin injection.

**FIGURE 1:**
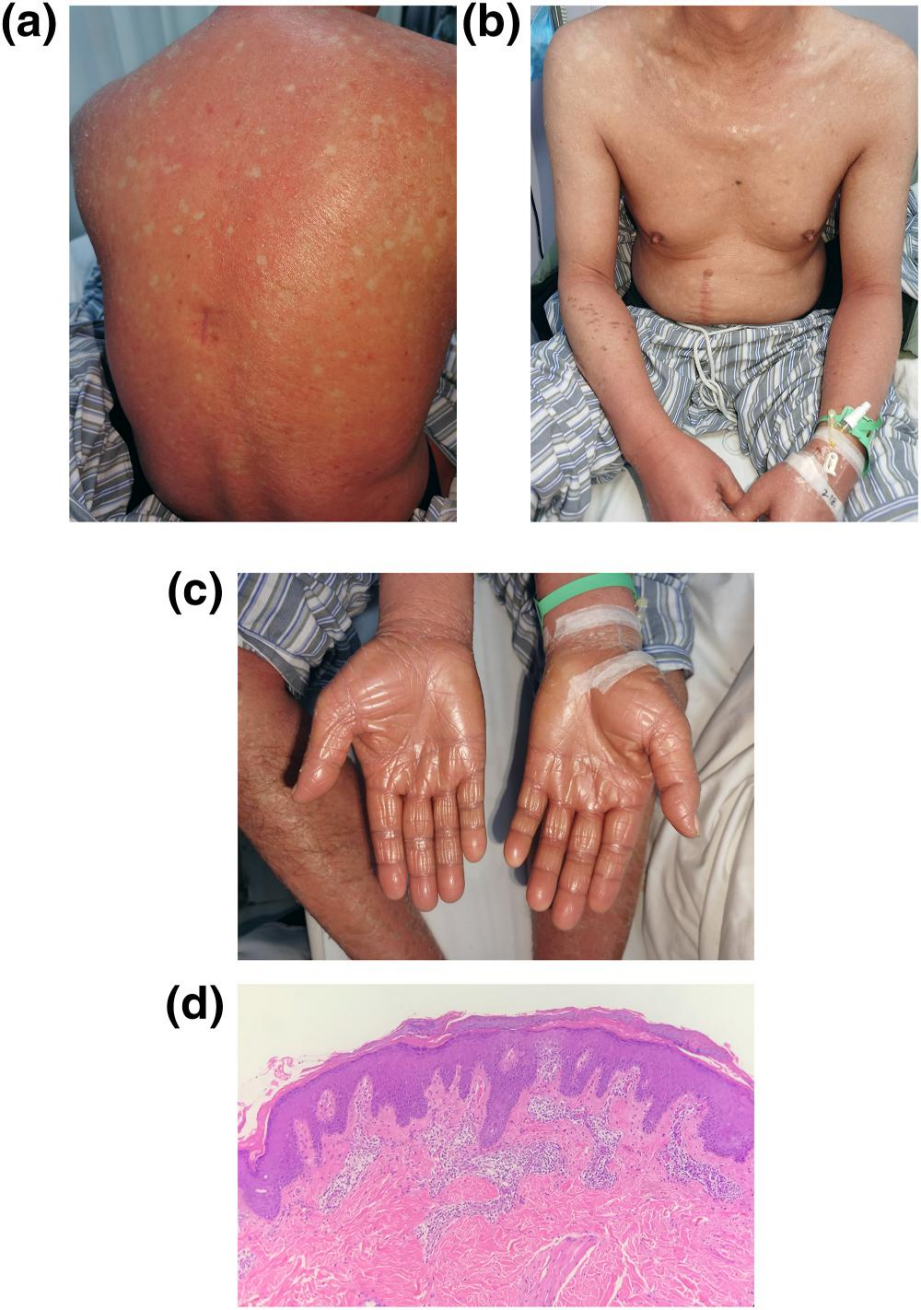
Clinical and histopathological images of the Pityriasis rubra pilaris (PRP) patient before ixekizumab treatment. (a, b) Confluent erythematous eruption all over his body with fine diffuse scales; (c) Orange‐red waxy palmoplantar keratoderma; (d) Histopathologic images of lesional skin sample. The epidermis shows hyperplasia. Alternation of orthokeratosis and parakeratosis in vertical and horizontal direction. Perivascular infiltrate in the superficial dermis (haematoxylin‐eosin staining; ×100 magnification)

Physical examination revealed widespread confluent erythematous plaques with fine diffuse scales surrounded by islands of spared skin, both eyelids ectropion with incomplete closure, orange‐red waxy palmoplantar keratoderma. He complained of intense itching of the skin. Fasting blood glucose was elevated with 7.6 mmol/L. Other routine laboratory parameters were within normal limits. Serology for human immunodeficiency virus and hepatitis B and C was negative. A skin biopsy of the back showed hyperkeratosis, psoriasis‐like hyperplasia, alternations of orthokeratosis and parakeratosis in vertical and horizontal direction, keratin plugs in the follicles, and blood vessel dilation surrounded by lymphocytes in the papillary dermis(Figure 1). The diagnosis of PRP (Type I) was made based on clinical presentation and pathological findings.

Acitretin therapy was started at an initial dose of 20 mg per day together with topical clobetasol propionate 2 times/day. After 2 weeks, the patient had no improvement of the skin lesions and developed mild elevated alanine transaminase to 108U/L, intense pruritus made the sleeping difficult. Considering that the patient had a history of fatty liver and acitretin had the possibility of damaging liver function, treatment was changed to off‐label use of ixekizumab, an interleukin‐17A inhibitor, after ruling out potential infections and obtaining the patient's written consent. It was administered as a subcutaneous injection with a loading dose of 160 mg followed by 80 mg every 2 weeks, which is the same with used for psoriasis.

**FIGURE 2:**
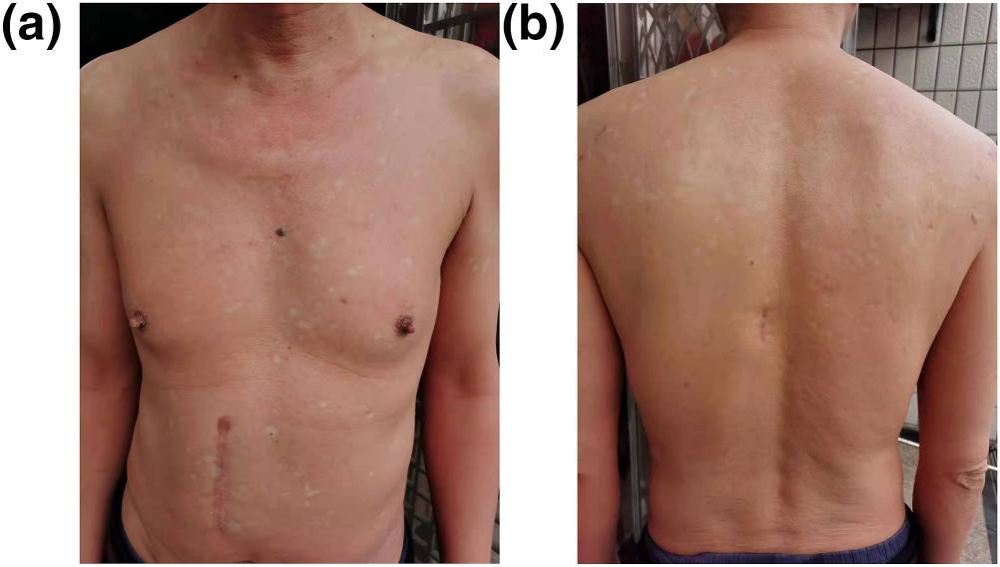
Clinical images of the Pityriasis rubra pilaris (PRP) patient 4 weeks after initiation of ixekizumab treatment

The patient exhibited rapid improvement. After 4 weeks of treatment, an improvement was achieved with decrease in both pruritus and erythema, and alanine transaminase was decreased to normal values (Figure 2). 12 weeks later, the skin had almost completely cleared of erythema and scaling with only 5% of body surface involved. Then the dosage changed to 80 mg every 4 weeks and discontinued after 5 months. The patient is still under follow up, no relapse and adverse effects has occurred.

## 3 DISCUSSION

In this paper, we present a case of PRP that developed after COVID‐19 vaccination and were successfully treated by Ixekizumab.

Currently, vaccination against SARS‐CoV‐2 is being carried out worldwide. The CoronaVac vaccine is a kind of inactivated COVID‐19 vaccine and is been widely used in lots of countries. Several cutaneous adverse effects after CoronaVac vaccination are currently being described such as chronic spontaneous urticaria/angioedema, contact dermatitis and anaphylaxis, but post‐vaccine PRP has been rarely reported in the literature.^2^, ^3^ Previous literature shows PRP can be induced by diphtheria‐tetanus polio, anti‐influenza and measles‐mumps‐rubella vaccines.^4^, ^5^ I Lladó et al and P.R. Criado et al showed 3 cases of PRP induced by adenoviral vaccine while M K Hunjan et al showed a case induced by mRNA vaccine.^6^, ^7^, ^8^ According to the course of our patient, the lesions were appeared after the first dose and significantly aggravated after the second dose of vaccination, we believe that the patient's PRP should be vaccine‐induced, although the association could be entirely casual.

Pityriasis rubra pilaris is a chronic papulosquamous disorder that can have a significant impact on quality of life and the treatment is challenging. Narrow Bound Ultra Violet B Light, isotretinoin, acitretin, methotrexate, cyclosporine can be used as therapy.^9^ Our patient was given acitretin initially but had no improvement and developed mild elevated alanine transaminase. Thus, we desired to apply biologic agents. Previous literature had already showed that Tumor Necrosis Factor inhibitors (Infliximab, adalimumab) and ustekinumab may be of value in treating PRP.^10^, ^11^ Feldmeyer et al found helper T cell 17 (Th17) profile, including IL‐17A, IL‐17F, IL‐22, were upregulated by mRNA expression analyses in skin biopsies of PRP lesions.^12^ Therefore, targeting IL‐17 may modulate the immune response, resulting in improved clinical outcomes for PRP. Several studies have showed that PRP cases have shown good response to secukinumab and ixekizumab.^13^, ^14^, ^15^ In these cases, secukinumab and ixekizumab were prescribed at the same dosage as that used for psoriasis. Dylan Haynes et al investigated the clinical response and safety of ixekizumab in treating PRP and found ixekizumab could reduce clinical signs and symptoms of PRP in several patients, including those in whom other systemic therapies have failed.^14^ Our patient achieved satisfactory efficacy after replacement to ixekizumab.

We report a new case of a PRP after COVID‐19 vaccination, and improved after a few weeks of treatment with ixekizumab. All in all, we should raise awareness of the skin adverse reactions to inactivated COVID‐19 vaccines, and anti‐IL17s appear to be a safe and effective therapeutic tool in PRP patients although larger, randomized, multicenter trials are still needed.

## AUTHOR CONTRIBUTIONS

**Ya‐ting Liu**: Data curation (Lead); Resources (Lead); Writing – original draft (Lead). **Shang‐shang Wang**: Conceptualisation (Lead); Formal analysis (Lead); Writing – review & editing (Lead).

## CONFLICT OF INTEREST

None to declare.

## ETHICS STATEMENT

We confirm that the study has been approved by research ethics committees and that appropriate consent has been obtained for studies involving human participants.

## Data Availability

The data that support the findings of this study are available from the corresponding author upon reasonable request.
